# Environmental Screening of Electrode Materials for a Rechargeable Aluminum Battery with an AlCl_3_/EMIMCl Electrolyte

**DOI:** 10.3390/ma11060936

**Published:** 2018-06-01

**Authors:** Linda Ager-Wick Ellingsen, Alex Holland, Jean-Francois Drillet, Willi Peters, Martin Eckert, Carlos Concepcion, Oscar Ruiz, Jean-François Colin, Etienne Knipping, Qiaoyan Pan, Richard G. A. Wills, Guillaume Majeau-Bettez

**Affiliations:** 1 Industrial Ecology Programme and Department of Energy and Process Engineering, Norwegian University of Science and Technology (NTNU), Sem Sælandsvei 7, 7491 Trondheim, Norway; guillaume.majeau-bettez@ntnu.no; 2 Energy Technology Group, Faculty of Engineering and the Environment, University of Southampton, University Road, Southampton SO15 1BJ, UK; awh1g10@soton.ac.uk (A.H.); rgaw@soton.ac.uk (R.G.A.W.); 3 DECHEMA-Forschungsinstitut, Theodor-Heuss-Allee 25, 60486 Frankfurt am Main, Germany; drillet@dechema.de (J.-F.D.); peters@dechema.de (W.P.); martin.eckert@dechema.de (M.E.); 4 Torrecid SA, Partida Torreta s/n, 12110 Alcora, Spain; carlos.concepcion@torrecid.com (C.C.); ORuiz@torrecid.com (O.R.); 5 Laboratoire des Matériaux, DEHT, LITEN, CEA, Université Grenoble Alpes, F-38000 Grenoble, France; jean-francois.colin@cea.fr; 6 Leitat Technological Center, Carrer de la Innovació, 2 08225 Terrassa, Spain; eknipping@leitat.org; 7 ACCUREC Recycling GmbH, Bataverstraße 21, DE-47809 Krefeld, Germany; qiaoyan.pan@accurec.de; 8 CIRAIG, École Polytechnique de Montréal, dép. Génie Chimique 3333 Chemin Queen-Mary, Bureau 310 C.P. 6079 succ. Centre-ville, Montréal, QC H3C 3A7, Canada

**Keywords:** Al-ion battery, rechargeable aluminum battery, electrode materials, anode material, cathode material, electrical energy storage, climate mitigation, environmental screening, sustainable development

## Abstract

Recently, rechargeable aluminum batteries have received much attention due to their low cost, easy operation, and high safety. As the research into rechargeable aluminum batteries with a room-temperature ionic liquid electrolyte is relatively new, research efforts have focused on finding suitable electrode materials. An understanding of the environmental aspects of electrode materials is essential to make informed and conscious decisions in aluminum battery development. The purpose of this study was to evaluate and compare the relative environmental performance of electrode material candidates for rechargeable aluminum batteries with an AlCl_3_/EMIMCl (1-ethyl-3-methylimidazolium chloride) room-temperature ionic liquid electrolyte. To this end, we used a lifecycle environmental screening framework to evaluate 12 candidate electrode materials. We found that all of the studied materials are associated with one or more drawbacks and therefore do not represent a “silver bullet” for the aluminum battery. Even so, some materials appeared more promising than others did. We also found that aluminum battery technology is likely to face some of the same environmental challenges as Li-ion technology but also offers an opportunity to avoid others. The insights provided here can aid aluminum battery development in an environmentally sustainable direction.

## 1. Introduction

The energy supply sector is the largest contributor (approximately 35%) to global anthropogenic greenhouse gas (GHG) emissions [1]. The continuous increase in demand for energy, depletion of nonrenewable resources, and concerns of climate change call for a change in our energy economy. Decarbonizing electricity generation is a key component of cost-effective mitigation strategies in reducing the sector’s high GHG emissions [1]. The primary candidates to replace fossil fuels are renewable energy sources [1,2]. Consequently, wind and solar power energy plants are attracting much attention [3]. However, the intermittence of these technologies requires efficient and economical electrical energy storage systems [3,4,5]. Rechargeable batteries, in particular, have been considered as a suitable alternative [4,6]. At present, Li-ion batteries are the prevailing choice for electrical energy storage due to their favorable characteristics such as long cycle life, low memory effect, high cycling efficiency, and high energy and power densities [3,6,7,8,9]. Even so, concerns regarding the high battery cost and the limited and geographically concentrated lithium reserves in the earth’s crust are driving research into alternative energy storage solutions [10,11,12].

Reaching beyond the horizon of Li-ion batteries is a formidable challenge that requires the exploration of new chemistry, especially electrochemistry, and new materials [13]. As a result, researchers are now looking for new battery chemistries dealing either with monovalent (K^+^, Na^+^) or multivalent (Mg^2+^, Ca^2+^, Al^3+^) cations [10]. Batteries based on multivalent ions are particularly attractive for large-scale energy storage applications because of their superior theoretical volumetric energy densities [11]. Among multivalent ions, the most promising cations for rechargeable batteries are abundant in the earth’s crust, light, and have small ionic radii, such as Mg^2+^ (0.72 Å) and Al^3+^ (0.53 Å) [11]. In spite of its high abundance in the earth’s crust, magnesium has a relatively high supply risk due to its geographically concentrated distribution [14]. In this regard, aluminum is a much more attractive alternative. Due to the low cost, low flammability, and trivalent ions, aluminum batteries can in principle offer cost-effectiveness, easy and safe operation, and high capacity [15,16,17]. Consequently, aluminum as an anode in primary Al-air batteries have long gained attraction, but these batteries have only found niche applications due to high rates of self-discharge and its nonrecharge ability in aqueous electrolytes, principally due to hydrogen gas evolution and aluminum passivation [18]. One possible strategy to avoid side reactions at the anode and cathode consists of coating both electrodes with an Al-ion conducting film such as Al_2_(WO_3_)_4_ [19]. Recently, the feasibility of secondary a Al-air battery with aprotic ionic liquids such as AlCl_3_/EMIMCl (1-ethyl-3-methylimidazolium chloride) have been demonstrated [20,21]. Nonetheless, most of these works were conducted in dry air to avoid both decomposition of extremely water-sensitive electrolytes and passivation of aluminum electrodes. Promising experiments in the less water-sensitive AlCl_3_/acetamide deep eutectic solvent have recently been reported in the literature [22]. Similar to the Li-air technology, development of a rechargeable Al-air system is very challenging and still relies on complex technologies for both air treatment and air electrode design, especially regarding triple-phase-boundary formation with a nonpolar electrolyte. In that context, the development of an Al-ion battery utilizing a room-temperature ionic liquid (RTIL) electrolyte and an insertion cathode material appears to be a more accessible target on the middle-term scale [15,16,17,23]. RTILs are suitable due to their relatively wide potential stability window and reasonable conductivity while being aggressive enough to remove any oxide layer from the surface of an aluminum electrode. However, RTILs are still expensive, corrosive, and often sensitive to moisture [24,25,26].

As very few results reporting on a rechargeable aluminum battery have been published up to now [27], efforts are still needed to find suitable electrode materials with satisfactory capacity, power density, and long-term stability. At this early stage of the technology, it is particularly important to evaluate environmental aspects of the electrode materials because the overall environmental performance of a final product is significantly influenced by choices made early in the product development [28]. Thus, an understanding of the environmental implications of the electrode materials is essential to make informed and conscious decisions in aluminum battery development.

The goal of this study was to evaluate and compare the environmental performance of various electrode material candidates for rechargeable aluminum batteries with an AlCl_3_/EMIMCl RTIL electrolyte. To this end, we used an environmental screening framework to evaluate the relative environmental performance of electrode materials considered within an Al-ion battery development project (ALION) and in the literature. By taking into account environmental concerns early in the battery development, we can guide aluminum battery development in a sustainable direction, thereby preventing lock-in effects and potential environmental pitfalls.

This article is structured as follows. In Section 2, we describe the method and the data used to evaluate the relative environmental screening. In Section 3, we present the results of the analysis. In Section 4, we discuss the results as well as their uncertainties and limitations and consider environmental aspects of aluminum battery production compared to Li-ion battery production. Finally, Section 5 concludes the study.

## 2. Materials and Methods

While lifecycle assessment (LCA) is a tool often used to evaluate the environmental impacts of products and technologies, it is not suitable for analysis in the early development stages of an emerging technology as data availability is insufficient [29,30]. Thus, instead we use Lifecycle Screening of Emerging Technologies (LiSET), an environmental screening framework [29], to evaluate the environmental characteristics of various candidate electrode materials. Rather than calculating the total potential lifecycle impacts of a technology not yet in existence, we propose the humbler (and more realistic) task of mapping out the likely relative strengths and the main areas of concern for each of the materials. Some of these areas of concern are extrinsic and may be overcome as technologies develop (e.g., energy requirements for material synthesis), while others are intrinsic and more fundamental (e.g., scarcity of certain elements), but all should be taken into consideration in prioritizing research efforts.

The determinants of the lifecycle environmental impact can be regrouped in four categories: (1) how many material inputs are needed for a technology to fulfill its function; (2) how environmentally intensive is the production of these materials; (3) how many energy inputs are required; and (4) how environmentally intensive is the production of this energy. The last element is very site dependent (local electricity mix, etc.) and not directly relevant to the design of a device that can be produced and deployed worldwide. This leaves us with three broad categories of data to map out a prospective technology’s environmental profile: material efficiency, environmental intensity of materials, and energy efficiency.

**Figure 1:**
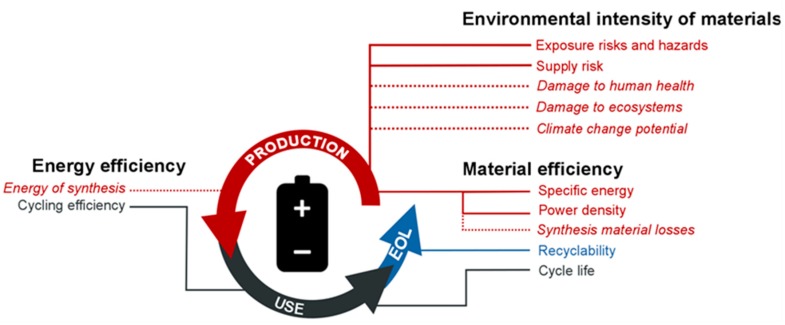
Lifecycle aspects of a stationary rechargeable battery. Solid lines denote intrinsic aspects of the material itself. Dotted lines and italic font denote extrinsic properties that are attributes of the value chain aspects or embodied activities related to the material’s production. Red lines denote production aspects, dark grey lines use phase aspects, and green lines end-of-life aspects. Abbreviation: EOL—end-of-life. Figure is adapted from [31].

In this article, we identified multiple aspects of an Al-ion battery’s production, use, and end of life that may serve as indicators of these three broad impact determinants (Figure 1).

The material efficiency is a metric of the functionality that a material can achieve per unit of mass. We focused on four intrinsic aspects—recyclability, specific energy, power density, and cycle life—and one extrinsic aspect—synthesis material losses.

The environmental intensity of material involves environmental aspects associated with a given mass of a certain material. We considered two intrinsic aspects—exposure risks and hazards during material handling and supply risk of a given material. As extrinsic aspects, we evaluated how the value chains producing these materials contribute to potential damages to human health, ecosystems, and climate.

Energy efficiency is a measure of how much functionality a given energy input can provide. Here, we considered one intrinsic aspect—cycling efficiency—and one extrinsic aspect—energy of synthesis.

Now that we have considered the various lifecycle aspects, we briefly describe the data that were used to evaluate the various aspects to yield a complete assessment of the different electrode material candidates; a more thorough account can be found in Appendix A. Quantitative data were preferred whenever available, but we also relied on qualitative and semiquantitative data. The exposure risks and hazards aspect was assessed based on the numerical rating of the Health aspect in the Hazardous Materials Identification System (HMIS) found in Material Safety Data Sheets (MSDS). Damages to human health and ecosystems were calculated with the ReCiPe method as integrated in the *ecoinvent* LCA database [32]. Climate change potential was calculated with the IPCC 2013 characterization factors with a 100-year time horizon. Supply risk was rated based on the socioeconomic availability dimension of SCARCE, a framework for the assessment of critical resource use [33]. The recyclability of the electrode materials was based on current battery recycling practices and on material properties. The specific energy evaluations were based on capacity and discharge voltage. The same performance data, in addition to specific current, were used to evaluate the power density. Cycle life and cycling efficiency ratings were evaluated based on cycle number, capacity loss, columbic efficiency, and voltage hysteresis. Given the early development stage of Al-ion chemistries, the relative importance of specific energy was lowered in comparison to specific power and cycle life. Volumetric energy density was omitted given the use of specific energy for the majority of current Al-ion material characterizations. Synthesis protocol data were used to evaluate both the synthesis material losses and the energy of synthesis. Even though various synthesis methods were used in the ALION project to produce the same materials, we only considered the material with superior electrochemical performance in this screening.

To make this relative assessment of 12 material candidates across 12 lifecycle aspects more easily grasped, we employed a three-category color code in a visual LiSET sustainability matrix: “green” for a perceived relative strength of a technology, “yellow” for intermediate characteristics, and “red” for an anticipated relative weakness. This format provides an at-a-glance assessment of how the different electrode materials perform as a whole (down column) and allows for easy comparison with other electrode materials (across columns).

## 3. Results

In this section, we go through the results of the environmental screening. Table 1 reports results for the evaluated electrode materials in terms of different intrinsic and extrinsic aspects that affect the environmental profile (environmental intensity of material, material efficiency, and energy efficiency)*.* While the “traffic-light” color grading indicates the perceived relative performance of the materials, a blank field indicates that the data availability was insufficient to evaluate a certain aspect for a given material. For materials where recycling is not a priority, due to abundant supply and no foreseeable shortage, the recyclability field is labelled N/A.

While anode materials are limited to aluminum-based materials, there are numerous alternatives for cathode materials, which is also reflected by the considered materials (Table 1). We find that no single material offers advantages in terms of all lifecycle aspects. There are, however, some materials that appear more promising than others. In the text below, we first consider the environmental characteristics of the anode materials and then the cathode materials.

### 3.1. Anode Materials

In AlCl_3_/EMIMCl electrolytes, the Al-ion battery is usually equipped with a pure aluminum foil in order to allow both kinetically fast Al stripping/deposition reactions and a larger voltage window in comparison with insertion anode materials. In this work, we considered a pure aluminum and an aluminum-titanate oxide insertion material. Between the two materials, there appears to be an environmental trade-off between environmental intensity and material and energy efficiency.

#### 3.1.1. Pure Aluminum

The low cost, high abundance, and high theoretical capacities (2980 mA·h·g^−1^ and 8040 mA·h ·cm^−3^) of the pure aluminum anode provide the primary motives for the development of Al-ion batteries. Another advantage is the low exposure risks and hazards associated with the pure aluminum anode. In spite of this, the aluminum anode has a relatively high environmental intensity because the high energy demand in aluminum refining causes high GHG emissions, which contributes to climate change potential as well as damages to human health and ecosystems [34,35]. On the other hand, aluminum is an abundant metal with high mining capacities, reserve concentrations, and low trade barriers, which contribute to low supply risk [33]. The pure aluminum anode offers high material efficiency. Even so, the columbic efficiency of current Al-ion cells should be carefully considered as aluminum stripping/deposition efficiency has been shown to vary with current density [36,37]. Variable columbic efficiencies of Al-ion cells at different cycle rates suggest the long cycle lives reported in the literature may be facilitated by the use of excess electrolyte and aluminum [17,23]. Improving the cycle life of Al-ion cells will of course improve the embedded lifetime energy, which should be a primary goal given the difficulty of surpassing the specific energy of current Li-ion chemistries. Generally, aluminum has high recycling rates and is also recovered in some industrial battery recycling processes [38]. The aluminum foil does not require any further processing than the supplier’s pretreatment, which we assumed to have low synthesis material losses and energy of synthesis. The low energy of material synthesis may offset some of the high energy demand in aluminum refining, along with the potential for efficient recycling at the end of the battery’s life. Finally, the pure aluminum anode displays relatively high cycling efficiency.

#### 3.1.2. Aluminum-Titanate Oxide Insertion Material

Compared to the pure aluminum anode, AlTiO_x_ presents somewhat higher exposure risks and hazards. AlTiO_x_ appears to cause intermediate damage to human health and ecosystems and has climate change potential. Similar to aluminum, titanium offers high abundance, mining capacities, and low trade barriers providing low supply risk for AlTiO_x_ [33]. The low environmental intensity of AlTiO_x_ is unfortunately not complemented with a high material efficiency. Instead, the material suffers from low specific energy, power density, and cycle life. In the ALION project, AlTiO_x_ nanopowder was synthesized through ceramic sintering and milling, resulting in a relatively high total material loss of 14%. In addition, the high energy of synthesis and unsatisfactory cycling efficiency resulted in relatively low energy efficiency.

### 3.2. Cathode Materials

For the cathode materials, we considered both insertion and conversion materials. While insertion materials allow metal ion intercalation/deintercalation, conversion materials undergo phase transition during charging/discharging steps. Due to their success in Li-ion batteries, a number of metal oxide insertion materials were tested in the ALION project. In addition, different types of carbonaceous insertion cathodes have been tested within the ALION project as well as in the literature. From the literature, we have additionally considered two insertion materials and one conversion material.

#### 3.2.1. Manganese Oxides

The high relative abundance and low supply risk of manganese makes the use of manganese oxides potentially attractive [33,39]. Here, we consider two manganese-based cathode materials: Co–MnO_2_ and K_0.2_MnO_2_. With the exception of supply risk, the two materials receive the same ratings for the environmental intensity aspects. The higher supply risk of Co–MnO_2_ compared to K_0.2_MnO_2_ stems from the use of cobalt [33]. With about 60% of the global production located in the Democratic Republic of Congo [40,41], cobalt is primarily mined as a coproduct and is subject to trade barriers, which particularly contributes to a high supply risk [33]. Co–MnO_2_ receives a high rating for recyclability as cobalt and manganese are currently recycled in commercial battery recycling processes [38,42,43], while K_0.2_MnO_2_ receives an intermediate recyclability ranking because potash minerals are irrecoverable and nonrecyclable [44]. Co–MnO_2_ and K_0.2_MnO_2_ produced initial capacities of 35 and 20–30 mA·h·g^−1^, respectively, at a 20 mA·g^−1^ cycling current. However, stable cycling could not be achieved for either of these manganese-based oxides and this resulted in poor cycle life. The two materials were produced through different synthesis methods. While ceramic sintering and ball milling used to produce Co–MnO_2_ resulted in intermediate energy demand and material losses, the hydrometallurgical processing of K_0.2_MnO_2_ required lower energy use and had low material losses when a recycling loop was implemented in the synthesis. Thus, between these two manganese oxide materials, there is a relatively low difference in the intrinsic aspects, but there is a trade-off in benefits and disadvantages of the different value chains.

#### 3.2.2. Other Transition Metal Oxides

Neither TiNb_2_O_7_, WO_3_, nor V_2_O_5_–B_2_O_3_ appear to be very promising cathode materials for the Al-ion battery. Of all the considered electrode materials, TiNb_2_O_7_ has by far the highest supply risk. Niobium is extremely unevenly distributed, with 90% of global production taking place in Brazil [41], and its availability is particularly subject to trade barriers [33]. TiNb_2_O_7_, WO_3_, and V_2_O_5_–B_2_O_3_ were capable of producing capacities of 48, 40, and 24 mA·h·g^−1^, respectively, at a 20 mA·g^−1^ cycling current, but the capacities decreased dramatically at higher specific currents, and a lack of extended cycling currently deems them unviable for use in Al-ion cells.

#### 3.2.3. Graphitic Materials

Battery-grade graphitic cathodes come in two main forms: natural graphite and synthetic graphite. While purified natural graphite used in batteries is extracted from mines, battery-grade synthetic graphites are primarily produced through chemical vapor deposition (CVD), where a gaseous carbon precursor, such as CH_4_, is processed at high temperatures [45,46]. In addition to CH_4_, renewable sources such as coconut oil and soybean oil can also be used as precursors [46,47]. Although synthetic and natural graphite both receive the same relative rating for climate change potential and damages to human health and ecosystems, synthetic graphite receives a higher quantitative score [34,35]. High EU import reliance, low substitutability, few major producing countries (about 70% of the global total is produced in China), high economic importance, and low recycling rates contribute to putting natural graphite on the 2017 EU list of critical raw materials [33,48]. Consequently, natural graphite receives a higher supply risk rating than synthetic graphite. Reducing the particle size from macro to nano increases the exposure risks and hazards of graphitic materials [49,50,51,52,53]. The various forms of carbonaceous cathodes have achieved good electrochemical performance. Capacities between 60 and 110 mA·h·g^−1^ have been obtained with cycle lives as high as 7500 [17,23,24,54]. Voltage efficiency can be estimated to be around 80% at low specific currents (<100 mA·g^−1^) for various graphites, although energy (voltage) efficiencies are rarely reported at higher specific currents. The greatest electrochemical performance difference between graphites is seen in the power capability. Natural graphite and especially graphitic foam perform considerably better than pyrolytic graphite at high specific currents [17,23]. Recently, graphene has also been utilized as a cathode, resulting in a good capacity of up to 120 mA·h·g^−1^ and extremely high cycle (>100,000) and power capabilities (100 A·g^−1^) [55]. The voltage profile produced by graphene is similar to those seen from other graphites. However, as with all Al-ion cells to date, low columbic efficiency at lower specific currents (as low as 80% at 100 mA·g^−1^) represents a serious drawback for real-world application as this affects the cycle life. In addition, graphite is also prone to delamination at high current densities and cycling number. The total capacity stored over the lifetime of an Al-ion cell with a long-life graphitic cathode could surpass many current Li-ion cells, excluding lithium-titanate batteries, although improvements to lifetime energy will be reduced due to the lower working potentials of Al/G cells of approximately 2.0 V. Nevertheless, operation at high specific currents could present an opportunity for Al-ion chemistries as power capability may become increasingly important for grid applications as renewable penetration increases [56]. Even though graphite is recoverable in theory, the graphitic anode material in Li-ion batteries is currently not recycled in practice but rather used to fuel the pyrometallurgical process in battery recycling [38,42,43]; it is likely that graphitic cathodes in Al-ion batteries will share the same fate. The lack of graphite recovery is primarily a supply concern for graphitic materials made from natural graphite rather than synthetic graphite. Mass-produced battery grade synthetic graphite is synthesized through thermal chemical vapor deposition (CVD), in which purified gases are processed at elevated temperatures (typically around 1000 °C) over a prolonged period [46]. While CVD generally tends to be energy intensive due to high temperatures, it has low material losses [57] and the production yield can be as high as 100% for graphitic materials [58].

#### 3.2.4. Conductive Polymers

Reversible doping of conductive polymers by chloroaluminate ions could allow their use in rechargeable aluminum batteries. Hudak examined conducting polymers (i.e., pyrrole, thiophene, polypyrrole powder, and poly(thiophene-2,5-diyl) powder as cathode materials [59]. Pyrrole and thiophene are associated with high exposure risks and hazards but are unlikely to be subject to supply risk. Because we were unable to obtain information about the synthesis and value chains of any these polymers, the affected lifecycle aspects were left unrated. As for the electrochemical performance, reasonable capacities between 30 and 80 mA·h·g^−1^ have been achieved for up to 100 cycles. The higher capacities were also obtained by widening the operating voltage, but this resulted in lowered columbic efficiencies, and consequently, cycle life. Capacities were also obtained at low specific currents of 16 or 20 mA·g^−1^ resulting in poor power capability [59].

#### 3.2.5. Titanium Sulfide

The TiS_2_ cathode was used in the first rechargeable Li-ion batteries [60] and recent literature has considered it for Al-ion batteries as well [61]. Unfortunately, the material does not stand out as a promising candidate for Al-ion batteries. Geng et al. reported a capacity of 50–70 mA·h·g^−1^ at a low 5 mA·g^−1^ cycling current [61]. The low operating voltage of 1.0 V would further lower specific energy and power. TiS_2_ was produced through solid state reaction and ball milling [61]. While the synthesis yield was not provided by the authors [61], one of the ALION partners reported that the combined losses of larger synthesis batches can be below 5%. The relatively high synthesis temperatures over long durations and the poor cycling efficiency [61] raise concerns about the material’s energy efficiency.

#### 3.2.6. Sulfur

Analogous to the widely investigated Li-S chemistry, Al-S presents the possibility of significantly increasing specific energy through the use of a sulfur conversion cathode in place of an insertion material. The sulfur-based cathodes were not researched in the ALION project but have been considered in the literature [62,63]. Volume expansion and sulfur dissolution followed by partially irreversible polysulfide formation reduce cycle life, though up to 50 cycles have recently been demonstrated [62,63]. While the low conductivity of sulfur also necessitates the addition of conductive materials, we here limit the discussion of the environmental intensity and recyclability to the pure sulfur material. Supply of sulfur is unlikely to become an issue, as it is a readily available by-product. Therefore, we consider sulfur recycling irrelevant. Very high capacities in excess of 1000 mA·h·g^−1^ have been reported, however, low discharge voltages present significant challenges [62,63]. Furthermore, large voltage hysteresis leads to poor cycle life. Depending on the conductive materials and synthesis methods used in the sulfur composite electrode material, the sulfuric cathode can be synthesized with low energy use and seemingly low material losses [62,63].

## 4. Discussion

The goal of this study was to evaluate the relative environmental performance of various electrode materials to guide rechargeable aluminum battery development in an environmentally sustainable direction. To this end, various electrode materials were evaluated in terms of different lifecycle aspects.

### 4.1. Discussion on Aluminium Battery Electrode Materials

Due to the limited options for anode materials, we considered only two anode material candidates. Compared to AlTiO_x_, the pure aluminum anode receives a more favorable rating for material and energy efficiency. However, the high energy use in aluminum metal refining constitutes a potential issue for the sustainable production of the pure aluminum anode. On the other hand, the aluminum foil did not require much further processing and its synthesis had miniscule energy inputs. In contrast, synthesis of the AlTiO_x_ electrode material had high energy requirements. In this regard, the environmental screening has identified tradeoffs in energy use between the upstream processes and the material synthesis for the anode electrodes. At present, it appears that AlTiO_x_ is unlikely to compete with the electrochemical performance of the pure aluminum anode.

For the cathode, we evaluated both insertion and conversion materials. In general, the transition metal oxides, the conductive polymers, and TiS_2_ displayed relatively low material and energy efficiency. Lowering the dissociation energy for Al^3+^ species and increasing the potential stability window are prerequisites for boosting performance of transition metal oxides, highlighting the need for further electrolyte developments. However, of the materials considered in this study, graphite seems to be the most promising candidate, followed by sulfur. There are, however, some concerns for these two materials as well. For the graphitic cathode, the most significant issue to overcome is the high energy use in material synthesis. Producing synthetic graphite through less environmentally intensive methods than CVD can significantly reduce its environmental footprint. Natural graphite tends to provide better power density and appears to have lower potential damage to human health, ecosystems, and the climate than synthetic graphite. In Al-S batteries, the use of conductive materials has improved the poor power density of the sulfur-based cathode, but cycle life still poses a challenge. Cycle life is important because it determines how much use we can get out of a battery before it has to be replaced. In a hypothetical case where we have determined that the Al-S battery has a lower production impact than the Al-ion battery with the graphitic cathode, although it could over the total lifetime have the higher environmental impact of the two batteries if it requires replacement while the other does not. The necessary conductive material in the sulfur-based electrode was not considered for the cathode’s environmental intensity and recyclability. Future research efforts into the sulfuric cathode material should carefully consider what conductive materials are used to avoid compromising its low environmental intensity.

To better guide the battery development, we distinguished between intrinsic and extrinsic aspects. Extrinsic aspects, such as synthesis material losses, can be modified by the researcher through the choice of synthesis method, while intrinsic aspects, such as the scarcity of a material, cannot. Even so, extrinsic aspects pertaining to the environmental intensity of the material are difficult for the researcher to address, as these aspects are upstream in the material value chain. It is also important to keep in mind that the synthesis methods affect the material properties, including electrochemical performance. For example, TiNb_2_O_7_ was prepared through sol–gel processing followed by hydrothermal treatment and calcination, but preparation via a flame spray pyrolysis or ceramic sintering resulted in inferior electrochemical performance. A less environmentally intensive synthesis method may not always be the best method for a given material. Thus, we might have to compromise between the environmental footprint of a synthesis method and the electrochemical performance. Even so, the distinction of extrinsic and intrinsic aspects is useful as it clearly signals to the researchers, stakeholders, and policy makers what aspects can be modified and which ones cannot.

While the “traffic-light” format used here inherently communicates the relatively high uncertainty at the current state of the technology, we further discuss data uncertainty in our study here. The data and evaluation pertaining to the material synthesis is, in particular, a source of uncertainty in this study. While we had good synthesis data for the materials made within the ALION project, we only had secondary synthesis data and descriptions for electrode materials that were produced in other studies or premanufactured. Therefore, the evaluation of synthesis material losses and energy of synthesis are based on a combination of quantitative and qualitative data, and this introduces an uncertainty aspect. For the sake of transparency, we categorized the rating of the synthesis material losses and energy of synthesis in Appendix A. To estimate the potential damages of the various electrode materials to human health, ecosystems, and the climate, we used global production mixes of constituent materials. Had we, for instance, chosen aluminum produced with hydroelectricity in Norway rather than the global production mix, the pure aluminum anode may have received a more favorable rating in our screening. Using global production mixes was a deliberate choice to focus on the main message, namely, that there might be potential issues with the value chain of a given material.

### 4.2. Comparison with Li-Ion Batteries

Now that we have discussed the different materials, we consider the cradle-to-gate environmental impact of a Li-ion battery cell to evaluate how the aluminum (Al-S and Al-C) batteries will perform compared to its main competitor. The environmental impacts of producing a lithium nickel-manganese-cobalt oxide (NMC) pouch cell are primarily caused by the production chains of three key requirements: the manufacture of the cells, the positive electrode paste, and the negative current collector [64]. For 12 environmental impact categories, these three production chains combined contribute to 81–99% of the Li-ion battery cell’s total production impact [64]. Thus, comparing these three production chains with the analogous production chains in the aluminum batteries can provide insights to the relative environmental performance of the new battery chemistries.

The environmental impact of the first key requirement, cell manufacture, stems from high energy use. Energy demand in Li-ion cell manufacture stems particularly from dry-room operation and electrode production [64,65]. Similar to Li-ion cell manufacture, the aluminum cell manufacture places stringent requirements to the ambient conditions and cleanliness in production zones. Generally, the AlCl_3_/EMIMCl ionic liquid is used as the RTIL electrolyte in aluminum battery cells due to its ability to allow the reversible dissolution and plating of metallic aluminum at reasonably high columbic efficiencies. AlCl_3_/EMIMCl is highly hygroscopic and must therefore be handled in dry conditions (i.e., glovebox or dry room). With respect to electrode production, the aluminum battery is likely to apply a pure aluminum anode that will not be coated with an active electrode material and this lowers the energy demand compared to Li-ion electrode production. The aluminum anode also affects what electrolytes can be used in the aluminum battery. The reversible stripping and deposition of aluminum in Al-ion cells currently requires the use of highly corrosive chloroaluminate RTILs. This can lead to potential leakage and Cl_2_ formation, factors which must be considered from a safety and human exposure viewpoint [25]. While aqueous electrolytes may allow for high power, safe and nontoxic cells utilizing the high capacity of metallic aluminum are not feasible in aqueous electrolytes due to the potential of reversible deposition being below that of H_2_ evolution, resulting in lower capacities.

**Figure 2:**
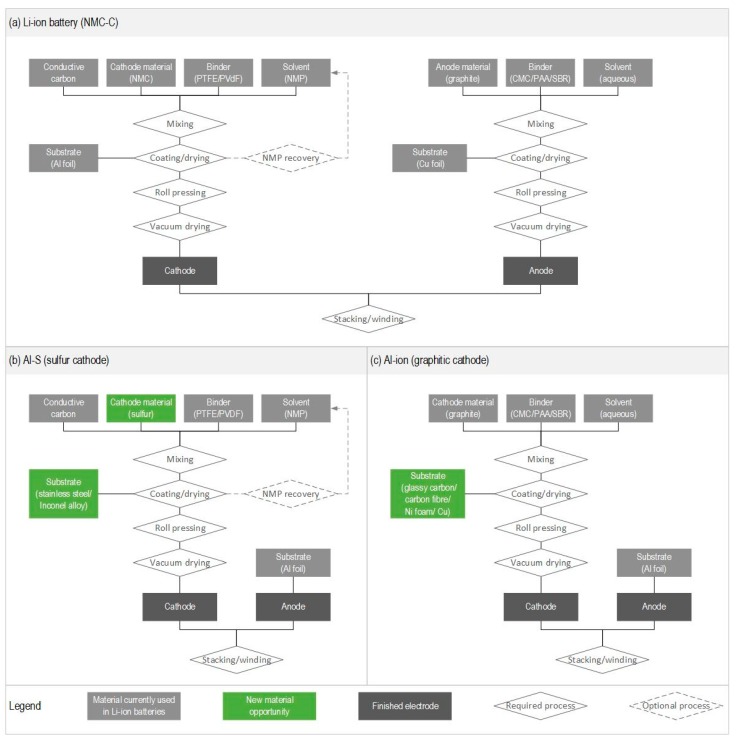
Electrode production requirements for (**a**) a Li-ion battery with NMC cathode and graphitic anode; (**b**) an Al-S battery with sulfur cathode and aluminum anode; and (**c**) an Al-ion battery with graphitic cathode and aluminum anode. Abbreviations: NMC—lithium nickel-manganese-cobalt oxide, PTFE—polytetrafluoroethylene, PVDF—polyvinylidene difluoride, NMP—*N*-methyl-2-pyrrolidone, CMC—carboxymethyl cellulose, PAA—polyacrylic acid, and SBR—styrene butadiene rubber.

The next two key requirements stem from electrode components, namely the positive electrode paste and the negative substrate [64]. Because the electrode production requirements differ for the Li-ion battery and the aluminum batteries (Figure 2), it is likely that the environmental impacts associated with these two key requirements will also differ.

The environmental impact from the positive electrode paste stems primarily from the positive active material (NMC), the polytetrafluoroethylene (PTFE) binder, and the *N*-methyl-2-pyrrolidone (NMP) solvent, while the conductive additive has insignificant contributions to the overall impact of the paste [64]. As seen in Figure 2a,b, the positive electrode paste based on NMC and sulfur both rely on PTFE or polyvinylidene difluoride (PVDF) as binders in combination with the NMP solvent [4,62]. Thus, for these positive electrode pastes, the main difference is likely to stem from the active materials (i.e., NMC and sulfur), while the binder and solvent materials are likely to be the same. In contrast and as illustrated in Figure 2c, the positive electrode paste in the Al-ion battery is more likely to rely on a graphitic active material with a water-based solvent in combination with binders such as carboxymethyl cellulose (CMC), polyacrylic acid (PAA), and styrene butadiene rubber (SBR). As illustrated in Figure 2a, this is the current production practice for the negative electrode paste in Li-ion cell manufacture [66]. Although modern Li-ion battery plants tend to recover the environmentally intensive NMP solvent after evaporation at a recovery rate around 96% [67], the NMP solvent demands more processing energy (heated air flow) to evaporate during electrode drying compared to water-based solvents [66,67]. In addition, PTFE and PVDF have been found to have a higher potential for climate change and ozone depletion than the binders used for the graphitic electrode material [64,68]. Thus, it may be beneficial to use synthesis methods that enable the exclusion of the PTFE and PVDF binders for the sulfur cathode.

Lastly, the environmental impact from the negative substrate in the Li-ion battery is largely caused indirectly through the disposal of sulfidic tailings in copper refining [64]. As illustrated in Figure 2b,c, studies researching aluminum conversion batteries with a sulfuric cathode have used stainless steel [62] and Inconel alloy [63] as substrates, while studies researching Al-ion batteries with a graphitic cathode have used glassy carbon [17,23], carbon fiber paper [24], nickel foam [55], and copper [54]. Because different substrate materials have different environmental impact, careful material selection can offer an opportunity to reduce the environmental footprint of the substrate in the aluminum batteries compared to the copper substrate in the Li-ion batteries.

Through this comparison, we find that the rechargeable aluminum batteries face some of the same environmental challenges as the Li-ion technology but also offer an opportunity to avoid others. Because the aluminum cell manufacture, like Li-ion cell manufacture, places stringent requirements on the ambient environment, we can expect considerable energy use and, consequently, environmental impact associated with the manufacture of rechargeable aluminum cells. Even so, the energy demands may be somewhat lower for the aluminum battery as it only requires paste application for one electrode. Furthermore, if the graphitic material is chosen, a water-based solvent, rather than the NMP solvent, can be used. This is likely to reduce the energy requirements in solvent evaporation and exclude energy needs associated with operating the NMP recovery unit. The sulfuric and graphitic electrode pastes can be paired with other substrate materials than copper and this presents an opportunity to reduce the environmental impact compared to Li-ion batteries.

## 5. Conclusions

Through this study, we have taken a cross-disciplinary approach to evaluate the relative environmental performance of various electrode materials for rechargeable aluminum batteries in combination with an AlCl_3_/EMIMCl RTIL electrolyte. We found that the aluminum anode and the graphitic or sulfuric cathode appear to be the most promising material candidates at present. However, these materials are associated with one or more drawbacks and therefore do not represent a “silver bullet” for the aluminum battery technology. Furthermore, the materials performing the best in this study may not be the ultimate electrode material candidates for rechargeable aluminum batteries; they were simply the best alternatives of the materials evaluated in this study. Even so, the screening performed here provides an early indication of the environmental benefits and disadvantages of a range of electrode materials. Furthermore, the considered environmental aspects and obtained results in this study offer checkpoints that can provide useful information for researchers developing new electrode materials for rechargeable aluminum batteries. In this way, the insights provided through the environmental screening and discussion can guide aluminum battery research in an environmentally sustainable direction and help avoid potential environmental pitfalls. This, in turn, can reduce the GHG emissions from the energy supply sector and result in important climate mitigation benefits.

## Figures and Tables

**Table 1:**
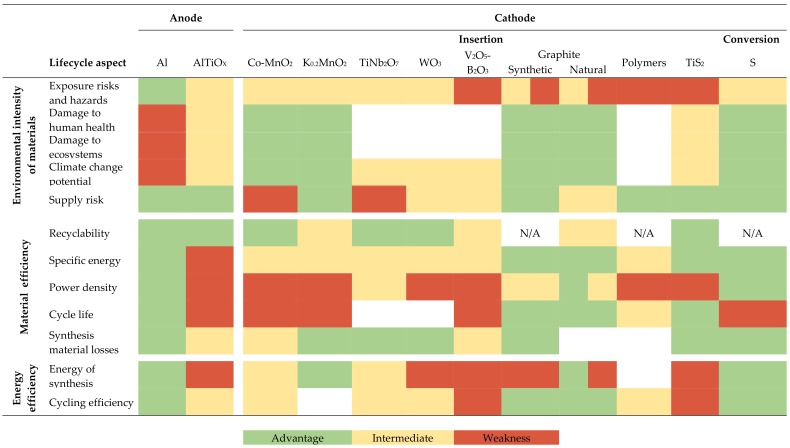
Relative environmental performance of electrode materials for rechargeable aluminum batteries with an AlCl_3_/EMIMCl electrolyte.

## Appendix A

### Appendix A.1. General Considerations for the Environmental Screening

For the evaluation of each lifecycle aspect, we strove to find a single, harmonized data source that would cover all technology candidates in a comparable manner. When this was not possible, fallback data sources were identified or, failing that, the data point was left blank in the LiSET matrix. Quantitative assessments were preferred whenever available, but this early development screening also included qualitative ranking, semiquantitative evaluations, and expert-judgement thresholds.

Because of the high uncertainty of such a screening, and to bring quantitative and qualitative scores on the same scale, all evaluations of lifecycle aspects were reduced to a lowest common denominator—a three-color scale. With this technique, we mark whether a given lifecycle aspect (e.g., cycling efficiency) constitutes a relative strength of a technology (“green”), a relative weakness compared to other technology candidates (“red”), or neither (“yellow”). This scale is relative: candidates are not ranked relative to an external standard of sustainability but rather ranked in proportion to the sample pool of materials under investigation.

In the case of quantitative evaluations of materials presenting a continuum of environmental performances (e.g., the global warming potential impact caused by the production of material), dividing the pool of technologies in three distinct groups (“green”, “yellow”, and “red”) represents a challenge. To minimize subjective judgement, the LiSET framework relies on a cluster analysis algorithm to regroup similar technology candidates into three groups. In the present case, the Jenks Natural Break Optimization was employed, as it is a clustering algorithm well suited to univariate data such as the one involved in our screening [69]. Less sophisticated alternatives to clustering algorithms include separating the sample pool into groups of equal size (e.g., quartiles or tertiles) or regrouping technologies the scores of which fall within equally sized ranges. Contrary to these methods, however, the Jenks Natural Break clustering algorithm generates groups that are as homogeneous as possible and places the boundaries between groups so as to avoid separating into different groups of data points that are close together at the border between clusters. These operations were performed relying on open-source Python modules, organized into a convenience LiSET tool (https://github.com/majeau-bettez/LiSET).

Lifecycle environmental performance indicators were rarely available for the exact chemical compounds under study but more frequently for the different metals that compose these compounds. In such cases, the score for the compound was calculated as a stoichiometric weighted average of its constituting elements.

We strove to apply the same assumptions and proxy choices for all technologies and materials. Consistency of comparison was prioritized over the accuracy of the representation of any individual technology. For example, environmental lifecycle impact data was available for the production of most metals in their reduced form but rarely for the production or extraction of their oxides. To ensure consistency of comparison, all metals were compared in their reduced form, even though metal oxides occur in many technology candidates. In all cases, we excluded from our analysis the impacts associated with binders, fillers, and additives to improve conductance, restricting our scope to the screening of the chemically active electrode material.

### Appendix A.2. Exposure Risks and Hazards

The exposure risks and hazards aspect was assessed based on the Health section of the Hazardous Materials Identification System (HMIS) rating found in Material Safety Data Sheets (MSDS). The Health rating uses a ranking system from 0 to 4, where 0 denotes no significant risk to health, while 4 denotes that life-threatening, major, or permanent damage may result from repeated overexposures. In this study, all materials had a Health rating of 3 or below. Materials with a Health rating below 1 were given a “green” rating, from 1 and below 2 were “yellow”, and 2 and higher were “red”.

HMIS Health ratings were available for the pure aluminum anode material and for some of cathode materials (i.e., tungsten trioxide nanopowder [70], pyrolytic graphite [53], graphitic foam [49], graphene oxide [52], and titanium sulphate [50]). However, for some electrode materials synthesized within the ALION project, MSDSs were unavailable. For these compounds, constituent material ratings were combined to make a stoichiometric weighted average (e.g., cobalt and manganese dioxide for cobalt-doped manganese dioxide). This was done for aluminum titanium oxide [71,72], cobalt-doped manganese dioxide [50,73], potassium manganese dioxide [50,74], titanium niobate [75,76], and vanadium pentoxide with boron trioxide [50]. Table A1 lists the rating for the various electrode materials and constituent materials.

**Table A1:** Exposure risks and hazards rating of materials.

Green	Yellow	Red
Aluminium	Aluminium titanium oxide	Cobalt
PVDF	Aluminium oxide nanopowder	Graphene oxide
	Boron oxide	Potassium
	Carbon foam	Pyrrole
	Manganese dioxide	Thiophene
	Niobium oxide nanopowder	Titanate sulphide
	Pyrolytic graphite	Vanadium pentoxide
	Titanium oxide nanopowder	
	Tungsten trioxide nanopowder	
	Sulphur	

### Appendix A.3. Potential Damage to Human Health, Ecosystems, and Climate from Material Production

One key determinant of a technology’s environmental performance is the level of environmental impact associated with the production of its constituting materials. These environmental impacts were assessed in terms of potential lifecycle damage to human health, ecosystems, and the climate. For most metals, lifecycle impacts were calculated with the attributional (“cut-off”) variant of *ecoinvent 3.4* database [77]. In all cases, global production mixes were used. Potential global warming impacts were calculated with a 100-year time horizon and with the IPCC 2013 characterization factors from the Fifth Assessment Report. Damages to human health and ecosystems were calculated with the ReCiPe characterization method as integrated in the *ecoinvent* database [32]. For cobalt, manganese, aluminum, and titanium, the reduced form of the metals were used. For boron and potassium, LCA scores were only available for the oxidized forms.

The *ecoinvent* LCA database had no data on vanadium, niobium, or tungsten, and their global warming potential impacts were instead taken from Nuss et al. [78]. The potential damages to human health and ecosystems of these three metals were not evaluated and left blank in the LiSET mapping matrix. We were not successful in finding LCAs of materials that could serve as good proxies for the specific polymers reported in the literature for Al-ion anodes. These were also left blank in the LiSET mapping matrix.

Because sulfur is obtained as a low-value by-product in fossil fuel and metal processing industries [79], we allocate no environmental burden to sulfur. This is in line with the surplus method—a partitioning method used in LCA that ascribes all impact to the primary product in a multiple output process [80].

### Appendix A.4. Supply Risk

To assess the supply risk associated with the different materials that make up the electrode materials, we relied on the Socioeconomic Availability Indicator of the SCARCE method, by Bach et al. [33]. This indicator takes into account concentration of reserves, company concentration, primary material use, feasibility of exploration projects, trade barriers, demand growth, concentration of production, price fluctuations, mining capacity, occurrence of coproduction, and political stability. It should be noted that the 2018 updated indicator scores were used (Daten für SCARCE Stand 01.2018) from the project website [81], rather than the scores originally published with the article.

Because supply risk scores span multiple orders of magnitude, the Jenks Natural Break clustering algorithm was applied on a log scale rather than on a linear scale. This prevents the two most problematic materials being the only ones classified as red and yellow, forcing every other material to appear green by their extreme value. Clustering the log of the values rather than the values themselves allows for the appreciation of important relative differences between smaller values.

Our scoring methodology initially planned for the application of a “double bottom-line”. In addition to the clustering algorithm, no material considered critical by the European Commission could be considered “favourable” (“green”) and would be forced to the next score (“yellow”). However, this corrective measure was not necessary since our initial clustering of the SCARCE scores already respected this rule.

### Appendix A.5. Specific Energy, Power Density, Cycle Life, and Cycling Efficiency

Performance aspects needed to be evaluated relative to a baseline electrode. Ultimately, a new battery chemistry must be compared to a suitable commercial counterpart, with Li-ion being state of the art for many applications. However, this was deemed unwarranted given the early stage of Al-ion development and potential differences in performance characteristics. Therefore, baseline characteristics of 1000 cycles and 60 mA·h·g^−1^ capacity when cycled at 100 mA·g^−1^ were chosen based on a mixture of Li-ion and current Al-ion cathode performance. Columbic efficiencies close to 100% are necessary for long cycle life, while cycling efficiencies above 80% are generally desirable. Similar performance to the baseline characteristics is indicated by “yellow”, improved performance by “green”, and inferior by “red”. The table below lists the high-level criteria used to evaluate the performance of materials. Table A2 sums up the criteria used to evaluate the specific energy, specific power, cycle life, and cycling efficiency of the evaluated electrode materials.

**Table A2:** Criteria used to determine performance of the electrode materials.

Specific Energy (mWh·g^−1^)	Specific Power (W·g^−1^)	Cycle Life and Cycling Efficiency
Capacity (mA·h·g^−1^)	Capacity (mA·h·g^−1^)	Cycle number
Discharge voltage (V)	Specific current (mA·g^−1^)	Capacity loss
	Discharge voltage (V)	Columbic efficiency
		Voltage hysteresis

### Appendix A.6. Recyclability

Evaluating the recyclability of the proposed Al-ion electrode materials is challenging, as many of these are novel electrode materials not used in batteries currently. Thus, we rated the recyclability of the electrode materials based on constituent elements. Where possible, we based the recyclability rating on current commercial battery recycling [38,43]. For elements that are not currently used in batteries, we used expert judgement based the material’s chemical properties to evaluate the recyclability. Currently, cobalt, aluminum, and manganese are recovered in commercial battery recycling processes [38,43] and received a “green” ranking. Based on their chemical properties, we deemed it likely that titanium, niobium, tungsten, and vanadium can be recovered from battery recycling. These metals also received a “green” rating. Even though graphite is recoverable, it is not recycled in any current commercial battery recycling processes [38,43]. Therefore, graphite received a “yellow” ranking. Because boron is difficult to reduce and likely to be lost in the slag in pyrometallurgical treatment, it was deemed challenging to recover and received a “yellow” ranking. Because potash minerals are irrecoverable and nonrecyclable [44], potassium received a “red” ranking. For materials where recycling is not a priority, due to abundant supply and no foreseeable shortage, the recyclability field was labelled N/A. We deemed that to be the case for synthetic graphite, sulfur, and polymers.

### Appendix A.7. Electrode Material Synthesis

This screening study evaluates a range of electrode materials, some synthesized specifically for the ALION project and some studied and prepared in previous publications. While many of the materials may be synthesized through various methods, we only evaluated the materials that had the best electrochemical performance within the ALION project and those reported in the literature. The text below describes the synthesis methods for the various electrode materials. First, we consider the materials that were synthesized within the ALION project. Then, we briefly describe the synthesis methods used in previous studies. After this, we consider the materials that were premanufactured. Finally, we categorize the synthesis material losses and energy of synthesis.

#### Appendix A.7.1. Materials Synthesized within the ALION Project

Aluminum titanium oxide, cobalt-doped manganese dioxide, and vanadium borate were synthesized through ceramic processing. To begin with, the raw materials were ball milled in ceramic mills to homogenize the mixture. Aluminum titanium oxide was obtained starting from AlOOH, rutile, and Li_2_O as doping agent. Cobalt doped manganese dioxide was obtained by the mixing of oxides, whereas vanadium pentoxide with boron trioxide was obtained by starting from boric acid and vanadium pentoxide. Afterwards, the mixtures were placed in refractory crucibles and sintered in ceramic furnaces with gas/oxygen burners. The sintering time and temperature varied for the different materials. Aluminum titanium oxide was sintered in a continuous furnace at 1100 °C for 4 h. Cobalt-doped manganese dioxide was sintered in a stationary furnace at 800 °C for 2 h. Vanadium pentoxide with boron trioxide was obtained as glass by melting at 800 °C for 2 h and air quenched. After sintering, the resulting powders were wet ball milled in an aqueous media, except for vanadium borate, which was jet milled. Cobalt manganese oxide was processed to 8 µm, while aluminum titanium oxide was subsequently milled in a micro bead mill to further reduce the particle size to 200 nm. After milling, the slurries were spray dried and recovered in filters. Energy consumption for the cobalt-doped manganese dioxide in the gas furnace was 4.7 KW/kg. The approximate material losses associated with the ceramic processing were 14% for aluminum titanium oxide, 12% for cobalt-doped manganese dioxide, and 17% for vanadium borate.

Potassium manganese dioxide was synthesized through several synthesis methods (i.e., solid state, sol–gel, hydrothermal, and nanosheet production) in the ALION project. The best electrochemical performance was found for potassium manganese dioxide on carbon black produced through hydrothermal synthesis. To begin the hydrothermal synthesis, KOH and KMnO_4_ were mixed together with a solvent consisting of water and isopropanol (20:1). The solution was stirred for 1 h at room temperature. After this, the mix was placed in a closed vessel for thermal treatment at 110 °C for 4 h. Finally, the mixture was cooled down and washed. While the energy requirements for this process were relatively low, the material losses were insignificant when a recycling loop was implemented.

Tungsten trioxide nanopowder was prepared through flame spray pyrolysis. Prior to starting, the flame spray pyrolysis equipment was heated up. The chamber was heated up to 900 °C for 1 h by feeding a solvent mixture into the flame and the filter unit was heated up to 200 °C to avoid water condensation. Then, tungsten alcoxide and solvents (made up hydrocarbons and organic acids) were combined together to a liquid mixture. After this, the liquid solution was fed into a burner nozzle in a fuel/oxygen mixture and dispersed at high speed into an oxygen-controlled flame where nanoparticles were formed and quenched on air. Afterwards, the nanopowders were separated from the air flow by filters and collected by inverse air flow in drums. The feeding rate was set to 350 mL/min, dispersion O_2_ 6 m^3^/h. The material losses of the process were approximately 8%.

Titanium niobate was synthesized through sol–gel processing followed by hydrothermal treatment and calcination. First, the titanium/niobium hydroxide precursors were mixed and stirred until the solution reached a pH of 10. Then, the mixture was transferred to a Teflon container and placed in a stainless-steel vessel for thermal treatment at 220 °C for 5 h. After this, the mixture was cooled down and washed with deionized water until it reached a pH of 6. The paste was dried at 60 °C overnight. Finally, the mixture was transferred to an alumina crucible and calcined at 750 °C for 1 h. While the synthesis material losses were not recorded for titanium niobate, we assumed low losses as one of the other ALION partners found insignificant losses when producing a cathode material using the hydrothermal method with a recycling loop and calcination.

#### Appendix A.7.2. Materials Synthesized in Previous Studies

A variety of graphitic materials have been investigated as cathodes in Al-ion batteries, including graphitic foam, pyrolytic graphite, natural graphite, and graphene. Where the material is novel, the details of synthesis are provided here, however, some publications rely on the use of commercially available graphite for cathode production.

Lin et al. [17] prepared graphitic foam through the chemical vapor deposition (CVD) method. Nickel foams were used as 3D scaffold templates for the CVD growth of graphitic foam. The nickel foams were heated to 1000 °C in a horizontal furnace under argon and hydrogen and annealed for 10 min. Then, methane was introduced into the reaction tube at ambient pressure and low flow rate, and after 10 min of reaction gas mixture flow, the samples were rapidly cooled to room temperature. The nickel foams now covered with graphite were drop-coated with a poly(methyl methacrylate) (PMMA) solution and then baked at 110 °C for 30 min. The PMMA/graphene/nickel foam structure was obtained after solidification. Afterwards, these samples were put into a solution for 3 h to completely dissolve the nickel foam to obtain the PMMA/graphite at 80 °C. Finally, the pure graphitic foam was obtained by removing PMMA in hot acetone at 55 °C and annealing in ammonia at 600 °C for 2 h, and then annealing in air at 450 °C for 2 h [17]. The authors did not specify their material losses or yield, but we assumed low material losses as CVD is said to have high production yields for graphitic materials [58,82].

A graphene film electrode, reported by Chen et al. [55], was synthesized by annealing a reduced graphene oxide (GO) film at 2850 °C for 30 min. The GO films were synthesized by cast coating or wet spinning liquid crystal graphene oxide onto another GO film [55]. A commercially available GO solution was used as the precursor. The modified Hummer’s method, reported by Marcano et al. [83], is a widely cited chemical route for the production of graphene oxide. Marcano et al. mixed graphite flakes, NaNO_3_, H_2_SO_4_, and KMnO_4_ and mixed the solution at 50 °C for 12 h before being cooled to room temperature for the addition of H_2_O_2_. The solution was filtered with the subsequent filtrate centrifuged then washed in water, HCl, and ethanol. The solid obtained after further centrifuging and filtering was vacuum-dried at room temperature. We were unable to make evaluations about the material losses for the graphene film.

Layered titanium sulphate was synthesized by Geng et al. [61] via solid-state reaction and ball milling. Here, we provide a short summary of the synthesis, and we refer the reader to the article by Geng et al. [61] for a complete description. Titanium and sulfur powders were thoroughly mixed and then sealed in an evacuated quartz tube, which was subsequently heated in a muffle furnace [61]. The temperature was first ramped up to 450 °C and held at this temperature for 24 h, after which the temperature was ramped up to 640 °C in 24 h and held at this temperature for 3 days [61]. After this, the synthesized titanium sulfur was collected and ball milled in an argon-filled glovebox. While the synthesis yield was not provided by the authors [61], one of the ALION partners reported that the combined losses for the solid-state reaction and ball milling of larger synthesis batches can be below 5%.

We considered two studies that had synthesized sulfur cathodes using different approaches. Cohn et al. [62] made a sulfur cathode by simply mixing together sulfur (50% by mass), Ketjen black carbon (30% by mass), and polyvinylidene fluoride (PVDF; 20% by mass). Gao et al. [63] fabricated an activated carbon cloth (ACC)/sulfur composite following protocols by Elazari et al. [84] and Kozen et al. [85]. Elemental sulfur was spread on the bottom of a stainless steel template and then the ACC was overlaid for preimpregnation with the sulfur and heated to 150 °C under slightly reduced pressure [84,85]. Subsequently, the material was sealed in a stainless steel vessel and further heated for at 155 °C for 10–15 h [84].

#### Appendix A.7.3. Materials Ready for Use

The pure aluminum foil considered as an anode material in the ALION project did not require any further treatment as the supplier had already pretreated the foil. To remove possible surface impurities, the supplier immersed the foil in an aqueous solution for 1–3 min at 60 °C [86]. We assumed no material losses connected to the pretreatment of the aluminum foil.

Hudak examined rechargeable aluminum batteries with conducting polymers (i.e., pyrrole, thiophene, polypyrrole powder, and poly(thiophene-2,5-diyl) powder) as cathode materials [59]. The polymers were used as received by the supplier [59]. Thus, no further energy or materials were used for material synthesis.

Pyrolytic graphite premanufactured by different suppliers was tested in the ALION project. Pyrolytic graphite was used as received from commercial sources. Commercially available pyrolytic graphite is produced by CVD at temperatures above 2500 °K or by the heat treatment of pyrolytic carbon, itself produced via CVD using a gaseous hydrocarbon, such methane, as in [45,87,88]. Pyrolytic graphite sheets can be produced from the heat decomposition of polymeric films at 1000 °K before graphitization at around 3000 °K and compression [89]. Similar to other graphitic materials synthesized through the CVD method, we assumed low material losses for pyrolytic graphite.

#### Appendix A.7.4. Synthesis Material Losses

For the synthesis material losses, we had some quantitative data from the ALION project, but we also had to rely on quantitative and qualitative data from the literature. Note that many of the materials required more than one of the synthesis methods. Treatment methods that had combined material losses below 10% were rated “green”, from 10% and below 20% were “yellow”, and 20% and higher were “red”.

#### Appendix A.7.5. Energy of Synthesis

Because quantitative data on energy requirements were not available for all materials, we evaluated the treatment methods based on semiquantitative data from the synthesis protocol descriptions. Table A3 presents the evaluations of the different methods. Note that many of the electrode materials required more than one treatment method. If a material required two treatment methods that had a “yellow” rating, this material would receive a “red” rating.

**Table A3:** Overview of energy of synthesis evaluations.

Green	Yellow	Red
Dry ball milling	Annealing (450 °C for 2 h and 600 °C for 2 h)	Annealing (2850 °C for 30 min)
Dry mixing of constituent materials	Calcination (750 °C for 1 h)	Ceramic sintering (1100 °C for 4 h)
Hydrothermal	Jet milling (75 min)	Chemical vapor deposition
Foil immersion in aqueous solution at 60 °C	Ceramic sintering (800 °C for 2 h)	Solid state with thermal treatment (450 °C for 24 h and 600 °C for 3 days)
Micro bead milling		Flame spray pyrolysis
Modified Hummer’s method		
Spray drying		
Wet ball milling		
